# Liquid Biopsy in Colorectal Cancer: Quo Vadis? Implementation of Liquid Biopsies in Routine Clinical Patient Care in Two German Comprehensive Cancer Centers

**DOI:** 10.3389/fonc.2022.870411

**Published:** 2022-05-12

**Authors:** Laura E. Fischer, Sebastian Stintzing, Volker Heinemann, Ulrich Keilholz, Dietmar Keune, Claudia Vollbrecht, Thomas Burmeister, Andreas Kind, Lena Weiss, David Horst, Thomas Kirchner, Frederick Klauschen, Andreas Jung, Christoph Benedikt Westphalen, Ivan Jelas

**Affiliations:** ^1^ Department of Medicine III, University Hospital, Munich, Germany; ^2^ German Cancer Consortium (DKTK), Partner Site Munich, German Cancer Research Center (DKFZ), Heidelberg, Germany; ^3^ Department of Hematology, Oncology and Cancer Immunology, Charité – Universitätsmedizin Berlin, Corporate Member of Freie Universität Berlin and Humboldt-Universität zu Berlin, Berlin, Germany; ^4^ German Cancer Research Center (DKFZ), German Cancer Consortium (DKTK), Partner Site Berlin, Heidelberg, Germany; ^5^ Comprehensive Cancer Center (CCC Munich LMU), LMU University Hospital Munich, Munich, Germany; ^6^ Charité Comprehensive Cancer Center, Universitätsmedizin Berlin, Corporate Member of Freie Universität Berlin and Humboldt-Universität zu Berlin, Berlin, Germany; ^7^ Institute of Pathology Charité – Universitätsmedizin Berlin, Corporate Member of Freie Universität Berlin and Humboldt-Universität zu Berlin, Berlin, Germany; ^8^ Labor Berlin – Charité Vivantes, GmbH, Molekulardiagnostik - Hämatologie, Berlin, Germany; ^9^ Institute of Pathology, Faculty of Medicine, Ludwig-Maximilians-University (LMU) Munich, München, Germany

**Keywords:** CRC, liquid biopsy, ctDNA, clinical practice, CGP

## Abstract

**Objectives:**

The use of liquid biopsies (LB) in patients with solid malignancies enables comprehensive genomic profiling (CGP) of circulating tumor DNA (ctDNA) and has the potential to guide therapy stratification and support disease monitoring. To examine clinical uptake of LB in a real-world setting, LB implementation was analyzed at two German cancer centers (LMU Munich and Charité - Universitätsmedizin Berlin) between 2017 and 2021, with focus on colorectal cancer (CRC) patients.

**Methods:**

In this retrospective analysis, all patients who received a LB between January 2017 and December 2021 as part of routine clinical management were included. To provide adequate context, we collected disease characteristics and technical specifications of the LB methods applied. Additionally, we examined the concordance of *RAS* status in tumor tissue and LB. Finally, we discuss the potential of LB as a diagnostic tool to drive personalized treatment in CRC patients and how to implement LB in clinical routine.

**Results:**

In total, our cohort included 86 CRC patients and 161 LB conducted in these patients between 2017 and 2021. In 59 patients, comparison between tissue-based and liquid-based molecular diagnostics, revealed a divergence in 23 (39%) of the evaluable samples.

**Conclusion:**

Our real-world data analysis indicates that the possibilities of LB are not yet exploited in everyday clinical practice. Currently, the variety of methods and lack of standardization, as well as restricted reimbursement for liquid based CGP hinder the use of LB in clinical routine. To overcome these issues, prospective clinical trials are needed to provide evidence driving the implementation of LB into the management of CRC patients and to support their implementation into clinical guidelines.

## Introduction

Worldwide, colorectal cancer (CRC) is a leading cause of cancer-related deaths. In metastatic CRC (mCRC), testing of biomarkers such as mutations in the *Kirsten rat sarcoma virus gene* (*KRAS), Neuroblastoma RAS gene (NRAS), v-Raf murine sarcoma viral oncogene homolog B1 gene (BRAF)* and microsatellite instability (MSI) is recommended and well established in clinical routine (1). However, tissue sampling does not always capture the full spatial and temporal genomic variability of CRC. Liquid biopsy (LB) is an emerging technology to detect and quantify cancer-specific genomic alterations in body fluids such as peripheral blood, ascites, urine or cerebrospinal fluid (2). The term LB encompasses techniques allowing the analysis of cell free deoxyribonucleic acid (cfDNA), circulating tumor DNA (ctDNA), circulating ribonucleic acid (ctRNA), proteins, cell-free or contained in circulating tumor cells, extracellular vesicles, or platelets. cfDNA derives among others from healthy cells of the body, a minor part of it is related to tumors and is called ctDNA. Genomic profiles of ctDNA have been shown to match those of the corresponding tumor tissue (3–6). Moreover, measurement of ctDNA has been used to predict disease outcome in gastrointestinal (GI) cancer patients (3) or help to define treatment strategy and duration (7, 8). Applications of LB in treatment of GI cancer are diverse and promising, however evidence for LB-driven therapeutic management of cancer patients is still limited.

In the treatment of mCRC, testing of genomic alterations in tissue are implemented in clinical routine (9). Additionally, other treatment relevant genetic alterations such as Neurotrophic tyrosine receptor kinase (*NTRK) gene* fusions and erb-b2 receptor tyrosine kinase 2 (*ERBB2)* amplifications/overexpression were discovered (10). For clinical research centers, multigene next generation sequencing (NGS) using gene panels has been implemented to simultaneously assess multiple clinical relevant biomarkers and to facilitate access to innovative clinical trials (10). Although there is ample published evidence proving the analytical and clinical validity of ctDNA in CRC, recommendations on the integration of ctDNA testing into clinical practice routine barely exist (11). Importantly, for further validation, ctDNA can be detected in nearly 90% of the patients with mCRC (12) and high concordance rates between LB and tissue are described (13). Data on the potential applications of ctDNA in mCRC are rapidly accumulating. For instance, ctDNA can be used to closely monitor the molecular evolution of epidermal growth factor receptor (*EGFR)* mutations in mCRC and identify patients who benefit from EGFR-specific antibody re-challenge (14).

Despite significant scientific advancements in the field of LB, broad clinical implementation of LB into routine clinical practice has not been achieved. Therefore, we performed a real-world data analysis, in which we focused on application and current clinical routine implementation of ctDNA testing in two German comprehensive cancer centers (University Hospital, LMU Munich and Charité – Universitätsmedizin Berlin) in CRC patients. In addition to describing the status quo, we aimed to identify the current challenges and discuss potential recommendations how to implement ctDNA measurement in clinical routine.

## Methods

In this retrospective data analysis, we included all CRC patients of the University Hospital, LMU Munich and Charité – Universitätsmedizin Berlin, who underwent LB between January 2017 and December 2021, as part of their routine clinical management. We collected data regarding methods, patients’ disease and the year the analysis took place. Furthermore, we evaluate genomic concordance of *RAS* status between LB and tissue biopsy.

From January 2017 until December 2021, 161 plasma samples taken from 86 patients were analyzed for ctDNA at University Hospital, LMU Munich and Charité - Universitätsmedizin Berlin (**Table 1**). 162 LB analyses were requested in routine clinical setting in cancer patients.

**Table 1:** Baseline characteristics of ctDNA analysis cohort of CRC patients.

Characteristic	Analysis set
Patients, n (%)	86 (100)
Charité - Universitätsmedizin Berlin	74 (86)
**University Hospital, LMU Munich**	12 (14)
**Site of primary tumor, n (%)**	
** Cecum carcinoma**	7 (8.4)
** Ascending colon**	9 (10.5)
** Right colic flexure**	1 (1.2)
** Transverse colon**	2 (2.3)
** Left colic flexure**	1 (1.2)
** Descending colon**	3 (3.5)
** Sigmoid colon**	28 (32.6)
** Rectum**	35 (40.7)
**Age in years, n (%)**	
** 31-40**	10 (11.6)
** 41-50**	19 (22.1)
** 51-60**	24 (27.9)
** 61-70**	17 (19.8)
** 71-80**	13 (15.1)
** 81-90**	3 (3.5)
**Gender, n (%)**	
** Male**	45 (52.3)
** Female**	41 (47.7)
**UICC Stage, n (%)**	
** I**	1 (1.2)
** II**	10 (11.6)
** III**	23 (26.7)
** IV**	52 (60.5)
** LB performed, n (%)**	**161**
** Successful**	132 (82.0)
** Failure**	29 (18)
** Pre-analytical**	19 (11.8)
** Analytical**	10 (6.2)
**Number of LB per patient, n (%)**	
** 1 LB**	65 (76.6)
** 2 LB**	10 (11.6)
** 3 LB**	2 (2.3)
** >5**	1 (1.2)
** >10**	4 (4.7)

n, number; UICC, The Union for International Cancer Control; LB, liquid biopsy.

meaning of bold, most important numbers, patient number and LB number.

At the Institute of Pathology, LMU Munich cfDNA was isolated from centrifuged plasma samples collected in Cell-Free DNA blood collecting tubes (BCTs,Streck, La Vista, NE, U.S.A.) (1. 10 min, 2800 x g, room temperature (RT); 2. 10 min, 6000 x g, RT) QIAamp Circulating Nucleic Acid Kits (Qiagen, Hilden, Germany) following Qiagen’s recommendations by performing a concentration step using a vacuum preparation station followed by an automated purification employing the Qiagen Cube. Nucleic acids concentrations were measured using Qubit v.3 fluorimetric technology (Thermo Fisher Scientific, Waltham, MA, U.S.A.) according to the handbook. Up to 60ng DNA (resembling about 10000 haploid human genomes) but in most cases less –as plasma samples contained low amounts of nucleic acids- was used as the input for NGS using either Oncomine Lung Cancer or Oncomine Colorectal Cancer Panels (Thermo Fisher Scientific) which were analyzed on an Ion Torrent GeneStudio S5 Prime (Thermo Fisher Scientific) or Archer nNGM Lung Cancer Variantplex Panels (Invitae, San Francisco, CA, U.S.A.) on an Illumina NextSeq 550 (Illumina, San Diego, CA, U.S.A.) device following the respective vendor’s recommendations. For reasons of sensitivity coverages of about 10000 reads were aimed for thus resembling ultra-deep sequencing.

At the Institute of Pathology Charité – Universitätsmedizin Berlin, blood samples were collected in cfDNA specific blood collection tubes (Cell-Free DNA BCT, Streck). cfDNA was extracted from double centrifuged plasma (10 minutes, 2000 g, RT; 10 minutes, 3000 g, RT) using magnetic bead based semi-automated Maxwell RSC instrument (Promega, Madison, WI, U.S.A.) after proteinase digestions (5% 20mg/ml Proteinase K (Promega) per milliliter plasma for 60 minutes at RT) according to the manufacturer’s instructions. Subsequently, cfDNA was concentrated *via* vacuum centrifuge (Concentrator plus; Eppendorf Hamburg, Germany) at 30°C for aqueous solutions, resolved in nuclease-free water. The total volume (up to 50 ng cfDNA) was subjected to library preparation with Oncomine Lung cfDNA Assay (Thermo Fisher Scientific). Final libraries were quantified using Ion Library TaqMan Quantitation Kit (Thermo Fisher Scientific) and sequenced on an Ion S5XL System (Thermo Fisher Scientific).

At Labor Berlin, the OncoBEAM RAS CRC Assay (Sysmex Inostics Inc, Baltimore, MD, U.S.A.) was used according to the manufacturer’s instructions. The test detects 34 different mutations in *KRAS* and *NRAS* codons 12, 13, 59, 61, 117 and 146. cfDNA was isolated from plasma samples collected in cfDNA specific blood collection tubes (Streck). Isolated cfDNA was pre-amplified with a multiplex polymerase chain reaction (PCR) and subsequently used in seven different emulsion (digital) PCRs for *KRAS* codon 12/13, *KRAS* codon 59/61, *KRAS* codon 117, *KRAS* codon 147, *NRAS* codon 12/13, *NRAS* codon 59/61 and *NRAS* codon 117/146. PCR products were bound to beads and hybridized with codon-specific probes, which were marked with different fluorescent dyes. The fluorescent beads were subsequently counted with a special flow cytometer (CyFlow Cube 6i, Sysmex Partec) using the CyViewTM software (Sysmex Inostics). Data were analyzed using the FCS Express software (V5.01 Sysmex Inostics).

Descriptive statistics were used to summarize the characteristic findings. Microsoft Excel for Windows 10, 2013 and SPSS version 28 for Windows (SPSS Inc, Chicago, IL) was used for statistical calculation.

## Results

### Number of CRC Patients at University Hospital, LMU Munich and Charité - Universitätsmedizin Berlin

**Figure 1:**
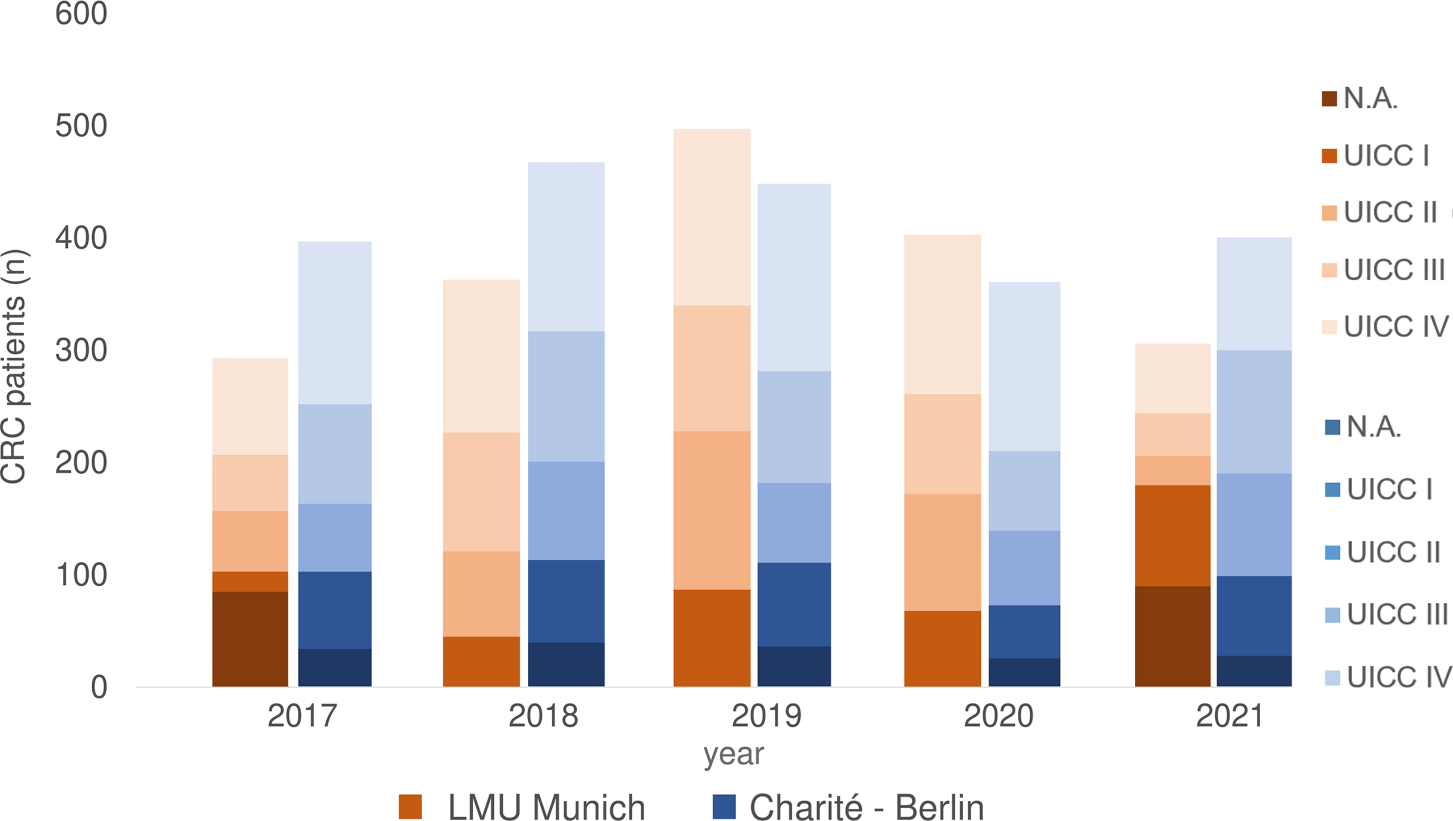
CRC patients 2017-2021 separated according to UICC stage at LMU Munich and Charite – Universitätsmedizin Berlin (including data from Labor Berlin – Charité Vivantes GmbH). N.A., not assessable.

From 2017 to 2021, 1818 CRC patients were treated at University Hospital, LMU Munich. According to Union for International Cancer Control (UICC) 264 patients had stage I, 401 stage II, 395 stage III and 583 a metastatic disease (stage IV). There was no data on tumor stage available for 175 patients (**Figure 1**).

At Charité - Universitätsmedizin Berlin, 2109 CRC patients were treated from 2017 until 2021, of which 341 had UICC stage I CRC, 381 UICC stage II, 492 UICC stage III and 723 UICC stage IV. There was no precise information on tumor stage for 172 patients (**Figure 1**).

### Real-World Data of ctDNA Analysis of CRC Patients

**Figure 2:**
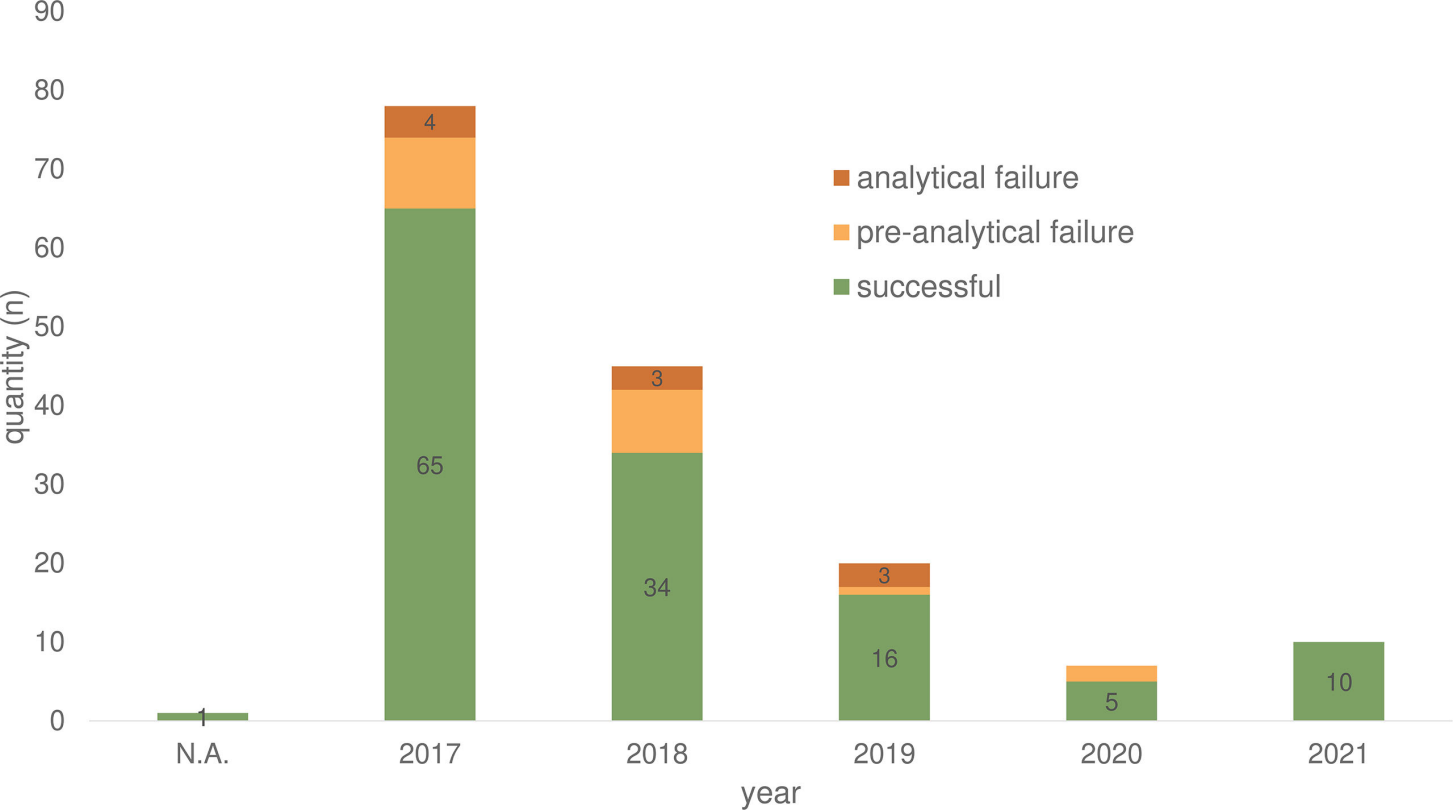
Plasma samples for ctDNA analyses of 86 CRC patients in the period from 2017-2021 divided according to success rate. N.A., not assessable.

In total of 132/161 (82.0%) ctDNA analyses of 86 patients were performed without failure (both cancer centers) (**Table 1**). Unsuccessful LB testing was grouped in pre-analytical issues (material insufficiency/n= 19; 11.8%) and analytical issues (technical problems/n= 10; 6.2%). The maximum number of conducted LB per year was registered in 2017, with a decline over the following years (2018 to 2020) registered for both sites (Berlin and Munich) (**Figure 2**).

The majority of patients had rectal cancer (n=35, 40.7%), followed by the sigmoid colon (n=28, 32.6%). The median age at the diagnosis of the investigated population was 57 years (median age at diagnosis in Germany > 70 years for women and men (15),). In the overall cohort, 45 (52.3%) patients were male, 41 (47.7%) were female (**Table 1**).

Patient cohort consisted predominantly of patients with metastatic disease (n=52, 60.5 %), followed by stage III (n=23, 26.7%) and stage II (n=10, 11.6%) CRC patients.

The mean time between initial diagnosis and following LB analysis was 29 months. The majority of patients received one LB testing, four patients received multiple LB analyses (**Table 1**).

In 65 patients, tissue *RAS* (*KRAS* and *NRAS* exons 2, 3, and 4) status was available and a comparison between tissue-based to liquid-based molecular diagnostics could be performed. In 23 patients (35.4%), we found discordant results of initial tissue based molecular analysis and future performed LB (**Table 2**). In none of the 23 patients the LB results influenced further treatment strategies. The lack of evidence of *RAS* mutations during tumor treatment was detected in 15 of the 65 patients (23.1%). The development of a new *RAS* mutation after anti-EGFR treatment was detected in 8 patients of 29 patients who received a LB after anti-EGFR treatment (27.6%). Retrospectively, different motives for LB testing were deduced. In 35 patients, LB was used to discover additional treatment options. In 25 patients, detection of minimal residual disease (MRD) was the reason for LB testing. 26 patients received LB testing to uncover resistance mechanisms.

**Table 2:** Divergence rate of RAS status in tissue and LB.

Characteristic	Analysis set
**Molecular diagnostics tissue, n (%)**	
***RAS* WT**	33 (38.3)
***RAS* MT**	38 (44.2)
**N.A.**	15 (17.4)
**UICC I/II/III**	13 (15.1)
**N.A.**	2 (2.3)
***RAS* status LB, n (%)**	132*
***RAS* WT**	77 (58.3)
***RAS* MT**	54 (40.9)
**Patients with available tissue and LB data, n (%)**	**65 (75.6)**
**Patients with tissue/LB divergence, n (%)**	23 (35.4) of 65 pts
**Loss of *RAS* MT, analysed by LB, n (%)**	15 (23.1)
**Patients treated with anti-EGFR therapy, n**	29
**Resistance *RAS* MT, n (%)**	8 (27.6) of 29 pts

n, number; RAS, rat sarcoma; WT, wild-type; MT, mutant; N.A., not assessable; LB, liquid biopsy; pts, patients. *One patient was ctDNA negative and had no evidence of disease.

meaning of bold, most important numbers, patient number and LB number.

Because of lack of evidence of ctDNA, two patients of 52 with stage IV disease deemed tumor-free and haven’t received additive tumor treatment after resection of liver metastases and no signs of tumor activity in the computed tomography (CT) scans. In one patient, LB verified a *BRAF* p.V600E after five months of initial tissue-based evidence of *BRAF* p.V600E. After appearance of new metastatic lesions, the patient was treated according to the BEACON CRC study (16) with encorafenib, binimetinib, and cetuximab.

## Discussion

The present analysis of real-world LB data was motivated by the limited clinical data regarding the implementation of LB in routine clinical CRC patient care in Germany. The aim of our analysis was to perform a reality check of LB utilization and to investigate the current value and to identify limitations of LB use. To our knowledge, this is the first publication of a real-world LB dataset of two independent German comprehensive cancer centers (University Hospital, LMU Munich and Charité – Universitätsmedizin Berlin) that is not clinical trial associated.

Over the past five years (2017 - 2021) 86 (2.19%) of the 3,927 (100%) treated CRC patients received one or more LB analyses. Especially in 2017, LB was used more frequently at Charité - Universitätsmedizin Berlin due to an early access diagnostic program for Labor Berlin that provided LB tests for free. At Labor Berlin, 86.3% of LB testing was performed covered by the early access program (139 LB/161 in total). Over the years (2018 - 2020), the data show a decline in the number of LB in both cancer centers (**Figure 2**). The lack of reimbursement and therapeutic consequences may have contributed to this result.

A discordance between tissue and liquid analyses could be determined in 23 (35.4%), of 65 patients (**Table 1**). Conversion from *RAS* wildtype (WT) to *RAS* mutated mCRC after treatment with anti-EGFR antibodies is a known and well-described acquired resistance mechanism with an estimated emergence rate in LB reaching 37% to 57% (17, 18). In our analysis, it was detected in 27.6% of the patients who received anti-EGFR treatment.

Interestingly, in 15 patients (23.1%) with initial detected *RAS* mutations (tissue) no evidence of them were observed in subsequent LB analysis. These cases have recently been termed Neo*RAS* WT (19, 20) and have rarely been described in the literature, potentially due to infrequent follow-up assessment of *RAS* mutated disease. The incidence of Neo*RAS* WT events (21) as well as effective treatments for these patients are unclear so far. Patients lacking the *RAS* mutation in LB may benefit from anti-EGFR rechallenge treatment (22, 23). Retrospectively, it remains unclear whether technical issues with respect to sensitivity had compromised these observations, regarding to correct number of detected Neo*RAS* WT cases in our cohort. Without knowing tumor specific mutations from the primary tumor as a reference, no clear statement concerning the *RAS* status in the LB can be made if no mutations are detected. The absence of mutations in LB analysis can be caused by too low amounts of ctDNA. Currently, it cannot be determined whether the patients ever shed ctDNA into the blood. Accordingly, a missing detection of *RAS* mutation, without another tumour specific mutation, cannot be seen as a Neo*RAS* status in certainty.

For treatment monitoring and tailoring, five patients with UICC IV CRC received five till ten LB during the course of the disease. Besides variations in the known *RAS* MT allele frequencies no further treatment relevant findings were observed. The short time interval (three weeks or shorter) between the performed LB could be on possibly explanation for the insufficient clinical benefit of the performed LB.

In general, our analysis showed a LB failure rate of 18% due pre-analytical (11.8%) and analytical issues (6.2%) (**Table 1**). Over the years, there has been a decline in the failure rate (**Figure 2**). Pre-analytical failure could be attributed to insufficient blood volume, hemolytic samples, transport issues or storage failure. Therefore, the implementation of LB into clinical routine needs to be accompanied by standardization processes, standard operating procedures (SOP), and training for both, the laboratory personal and the staff working with the patients (24).

Because of the improved pre-, and intra-analytical settings (e.g. transportation and storage of LB specimens) in clinical studies, the real-world data presented here cannot be compared directly with other published LB data with regard to failure rates.

To minimize failure rates, standardization of pre-analytical and analytic variables, including common methodology for blood collection and transport, plasma separation, storage and DNA extraction, platform methodology, breadth and depth of coverage, analytical validity and turnaround time must be optimized (25, 26).

The potential benefit of LB in CRC patient, was underlined in several preclinical and clinical studies. Focus of these investigations was the:

1. Early disease detection by LB (27–29);

2. Monitoring and treatment tailoring (30);

3. Monitoring of tumor response (31, 32);

4. Assessing of resistance mechanisms (33, 34);

5. Detection of minimal residual disease (MRD) in CRC (35, 36).

Based on the current published data and the here demonstrated retrospective real-world data of LB testing in two large German cancer centres, further prospective clinical trials, and the conception of LB specific evidence- based guidelines are warranted needed to clarify out the clinical impact and utility of LB in CRC patient care.

The small number of performed LB and the lack of methodical standardization between the two cancer centers made it impossible to perform ample statistical analysis and draw comprehensive conclusions for CRC clinical patient care. The absence of a clearly regulated cost coverage hinders a broader utilization in clinical patient care. The establishment of a structured reimbursement of LB for selected indications in CRC patients could stimulate further implementation of LB in routine patient care

## Conclusion

Liquid biopsy is a convenient, safe, and increasingly established method to analyze ctDNA from blood samples. Real world data of two independent comprehensive cancer centers in Germany covering the period of 2017-2021 show, that liquid biopsy is not implemented in routine clinical management of CRC patients, yet. Reasons are manifold but unresolved reimbursement issues, unsatisfactory data from clinical trials, and unclear validity of the clinical impact in different treatment situations needs to be solved. In our opinion, LB has high potential in CRC and beyond to inform and guide clinicians in the future with respect to resistance mechanisms, minimal residual disease and monitoring of treatment efficacy. Therefore, clinical trials are needed to provide the evidence and inform clinical guidelines before a broad clinical utilization of LB in CRC will take place.

## Data Availability Statement

The raw data supporting the conclusions of this article cannot be made available online. The raw data can be made available on request to qualified and authorized individuals in person.

## Ethics Statement

Ethical review and approval was not required for the study on human participants in accordance with the local legislation and institutional requirement.

## Author Contributions

LF, IJ: data analysis and interpretation, statistical analysis and paper writing. CV, DH, TB, TK, FK, AJ: Laboratory and/or morphological analysis of samples. CW, SS, TK, DH, FK, VH, UK, DK, AK: data interpretation and paper writing. AJ, CV, LW: data collection and paper writing. All authors contributed to the article and approved the submitted version.

## Conflict of Interest

Author TB was employed by Labor Berlin – Charité Vivantes GmbH.

The remaining authors declare that the research was conducted in the absence of any commercial or financial relationships that could be construed as a potential conflict of interest.

## Publisher’s Note

All claims expressed in this article are solely those of the authors and do not necessarily represent those of their affiliated organizations, or those of the publisher, the editors and the reviewers. Any product that may be evaluated in this article, or claim that may be made by its manufacturer, is not guaranteed or endorsed by the publisher.
